# Overview of Ca^2+^ signaling in lung cancer progression and metastatic lung cancer with bone metastasis

**DOI:** 10.37349/etat.2021.00045

**Published:** 2021-06-28

**Authors:** Manh Tien Tran

**Affiliations:** Department of Dental Pharmacology, Graduate School of Medicine, Dentistry and Pharmaceutical Sciences, Okayama University, Okayama 700-8525, Japan; The University of Texas at Arlington, USA

**Keywords:** Lung cancer, Ca^2+^ signaling, bone metastasis, osteoclasts, bone microenvironment

## Abstract

Intracellular Ca^2+^ ions that are thought to be one of the most important second messengers for cellular signaling, have a substantial diversity of roles in regulating a plethora of fundamental cellular physiology such as gene expression, cell division, cell motility and apoptosis. It has been suggestive of the Ca^2+^ signaling-dependent cellular processes to be tightly regulated by the numerous types of Ca^2+^ channels, pumps, exchangers and sensing receptors. Consequently, dysregulated Ca^2+^ homeostasis leads to a series of events connected to elevated malignant phenotypes including uncontrolled proliferation, migration, invasion and metastasis, all of which are frequently observed in advanced stage lung cancer cells. The incidence of bone metastasis in patients with advanced stage lung cancer is estimated in a range of 30% to 40%, bringing about a significant negative impact on both morbidity and survival. This review dissects and summarizes the important roles of Ca^2+^ signaling transduction in contributing to lung cancer progression, and address the question: if and how Ca^2+^ signaling might have been engaged in metastatic lung cancer with bone metastasis, thereby potentially providing the multifaceted and promising solutions for therapeutic intervention.

## Introduction

Intracellular Ca^2+^ [(Ca^2+^)_i_] signaling is implicated in regulation of a variety of physiological processes deciding either cell survival or death. In unexcited states, (Ca^2+^)_i_ ions are maintained at a very low level (in a range of 50–150 nM) nonetheless, a transient elevation of (Ca^2+^)_i_, obtained through either (Ca^2+^)_i_ efflux from intracellular organelles into cytosol or through (Ca^2+^)_i_ influx into cytosol from extracellular milieu, could mediate activation of various downstream signaling cascades [1]. Specifically, (Ca^2+^)_i_ fluctuation is tightly regulated by a series of Ca^2+^ channels, pumps and/or exchanges. Dysregulation of (Ca^2+^)_i_ homeostasis is a cause of the certain diseases such as developmental disorders, hypertension, cardiovascular disease, diabetes, Alzheimer’s disease, and cancer [2, 3]. In the context of cancer, whether dysregulated (Ca^2+^)_i_ homeostasis is necessary for malignant initiation has been disputable; however, there have been increasing cues proposing that dysregulation of (Ca^2+^)_i_ homeostasis might be a central point of defects in mechanisms upon tumor promotion.

The Ca^2+^ channels, pumps and/or exchanges at plasma membrane (PM) predominantly mediate the intermittent Ca^2+^ flux from outside to inside of cells such as voltage-gated Ca^2+^ channels (VGCCs), specific receptor-operated channels (ROCs) and store-operated Ca^2+^ channels (SOCs), which are stimulated by membrane depolarization, by the external agonists and by depletion of internal Ca^2+^ stores, respectively. Meanwhile, the inositol-1,4,5-trisphosphate (IP_3_) receptor (IP_3_R) and the Ryanodine receptor (Ca^2+^-induced Ca^2+^ release channels-RyR) are two key Ca^2+^ receptors releasing Ca^2+^ from the internal stores such as endoplasmic reticulum (ER). Mechanistically, binding of IP_3_ ligand to IP_3_R triggers IP_3_R activation, resulting in Ca^2+^ release from ER into cytosol [4] whereas RyRs, whose activity is dependent upon (Ca^2+^)_i_ concentration, release (Ca^2+^)_i_ from ER into cytosol in different cell types such as neurons, muscle cells, and epithelial cells [5, 6]. In addition, there are two leading systems responsible for Ca^2+^ extrusion across PM, including (1) the plasmalemmal Ca^2+^-ATPase (PMCA), a calmodulin (CaM)-dependent Ca^2+^ ATPase regulating contractility in vascular, bladder and uterine smooth muscle [7], and (2) the electrochemically driven Na^+^/Ca^2+^ exchanger (NCX), which is a bi-directional transporter exchanging three Na^+^ for one Ca^2+^ critically regulating (Ca^2+^)_i_ in heart [8]. Besides, (Ca^2+^)_i_ accumulation into ER could be mediated by the sarco-ER Ca^2+^-ATPase (SERCA), ubiquitously present in ER of all eukaryotic cells. For instance, SERCA played a role in promoting relaxation via pumping (Ca^2+^)_i_ into the lumen of sarcoplasmic reticulum (SR) that is a major subcellular pool of Ca^2+^ [9].

Lung cancer, also known as lung carcinoma, is the most frequently diagnosed malignancy and the leading cause of cancer death globally. Two major types of lung cancer best characterized include small cell lung carcinoma (SCLC) and non-SCLC (NSCLC), the latter accounting for approximately 85% of all lung cancers spreads locally to the thoracic cavity and to distant organs including bone [10]. Specifically, a range of 30%–40% of patients diagnosed with advanced stage lung cancer might have developed bone metastasis in a course of their etiological progression, bringing about a significant negative impact on both morbidity and survival [11]. Neoplastic bone formation is primarily derived from dysregulation of bone remodeling and homeostasis, tightly controlled by two functionally interrelated types of cells, (1) osteoblasts (OBs), which account for bone formation and (2) osteoclasts (OCs), which are responsible for bone resorption. It has been demonstrated that the “horrific consequence” of bone metastasis occurs as metastatic cancer cells enable to stimulate bone-resorbing activity of OCs, thereby leading to enhanced bone resorption [12]. Importantly, Ca^2+^ ions and cytokines released from osteoclast-triggered bone resorption promote tumorigenesis, contributing towards augmentation of tumor-propagating capacity of cancer cells and osteoclast-triggered bone resorption as well [13].

In summary, understanding of causes and consequences of regulatory mechanisms of (Ca^2+^)_i_ signaling associated with lung cancer progression and development of metastatic lung cancer with bone metastasis may shed a light on the potential therapeutic targets or prognostic biomarkers for treatment of lung cancer patients with advanced stage lung cancer with bone metastasis.

## Dysregulated Ca^2+^ homeostasis and lung cancer progression

As abovementioned, whether or not homeostatic disturbance of (Ca^2+^)_i_ signaling, either transient or sustained, is one of major causes to initiate malignant events, comprising cell cycle, apoptosis, and metastasis, is still questionable. Nevertheless, followed by such malignant events, dysregulated (Ca^2+^)_i_ signaling is frequently observed to contribute towards tumor progression. In this review article, the in-depth mechanisms upon contribution of dysregulated (Ca^2+^)_i_ signaling towards lung cancer progression as well as metastatic lung cancer with bone metastasis were discussed.

### The effects of dysregulated Ca^2+^ signaling on cell cycle

At early stage of tumor progression, cancer cells normally acquire a vast number of biological alterations that sustain their uncontrolled replicative capacity. Over last few decades, upon the development and technical modernization allowing to probe (Ca^2+^)_i_ transient oscillation. The functional importance of (Ca^2+^)_i_ signaling in regulation of cell cycle has been progressively unveiled. As a consequence of the greater than several thousand-fold gradient between (Ca^2+^)_i_ and extracellular Ca^2+^ [(Ca^2+^)_e_] levels [14], the opening of cell surface Ca^2+^ channels leads to an immediate influx of (Ca^2+^)_e_ across PM. Besides, the transient elevation of (Ca^2+^)_i_ could be mediated through Ca^2+^ efflux from the internal Ca^2+^ stores such as ER, Golgi complex and the others.

Recently Ca^2+^ signals have emerged to be the hub of controlling G1 phase, G1/S and G2/M phase transitions [15]. In reality, cells are frequently sensitive to depletion of (Ca^2+^)_e_ in G1, in which Ca^2+^ is critical for the expression of specific genes required for cell division such as *FOS*, *JUN* and *MYC*. Specifically, FOSL1, also known as aka FRA-1, a member of Fos family, is required for Kras-induced lung tumorigenesis *in vivo*, and promotes human lung adenocarcinoma proliferation and survival [16]. Besides, C-myc functions as a downstream signal of several growth factor receptors such as epidermal growth factor receptor (EGFR), transforming growth factor alpha (TGFα), transforming growth factor beta (TGFβ) receptor, interleukin (IL)-6 receptor, Notch receptor, and Frizzled receptor [17]. Importantly, C-myc also serves as one of the master transcription factors of many target genes that encode for proteins essential for regulation of cell growth and proliferation such as p15, p21, CDK4, CDC25A, E2F1 [18–22].

One of most important pathways regulated by (Ca^2+^)_i_ signaling towards cell cycle progression is mitogen-activated protein kinase-renin-angiotensin system (MAPK-Ras) pathway. MAPK [rat sarcoma virus (Ras), rapidly accelerated fibrosarcoma (Raf) and mitogen-activated protein kinase (MEK)] pathway keeps a major role in regulating a variety of cellular processes such as proliferation, differentiation and surivial. The abnormal expression of MAPKs is frequently observed in NSCLC [23]. It is best characterized that MAPK pathway is initiated by the external stimuli such as hormones, growth factors (GFs), cytokines and intracellular molecules, following the activation of the RAS upstream receptors including receptor tyrosine kinases (RTKs) and EGFRs. Activation of MAPK-Ras signaling pathway promoting cell cycle progression [24] by retinoblastoma (RB1) phosphorylation, which then triggers upregulation of cyclin D1-induced CDK4 or CDK6, eventually driving G1-to S-phase transition [25–32]. Deregulation of EGFR, also called ErbB-1, was found in a range of 40–89% of NSCLC [33]. Furthermore, (Ca^2+^)_i_ is also crucial for regulation of several Ca^2+^-dependent cascades such as calciuneurin (CaN) and CaM-kinase. CaN, a Ca^2+^- and camodulin-dependent serine/threonine protein phosphatase, plays a key role in promoting cell cycle progression at G1/S phase transition through cyclin D1 stabilization [34]. Liu et al. [35] identified that CaNAα, an isoform of CaN, which was overexpressed in lung cancer tissues, promoted cell proliferation through accelerating G1-to S-phase transition in SCLC cells *in vitro*. CaN inhibition by cyclosporin A (CsA) blocked the transcriptional activity of CREB binding protein (CBP) and the nuclear factor of activated T cells (NFAT), leading to alleviate the expression of pro-inflammtory cytokine genes [36, 37]. Noticeably, activation of transcription factors such as CREB and myocyte enhancer factor-2 (MEF-2) was regulated by (Ca^2+^)_i_ elevation by the (Ca^2+^)_e_ influx across PM via L-type voltage-gated channels (LTCs) [38]. CsA-triggered CaN inhibition declined CDK2 activity by diminishing the expression of cyclin D1 during G1 [39], cyclin E and cyclin A [40]. Increases in (Ca^2+^)_i_ concentration result in activation of CaN, which subsequently dephosphorylates NFAT proteins, allowing them to translocate to the nucleus to regulate the expression of the target genes [41].

Orai3 channels, frequently overexpressed in NSCLC, mediate Ca^2+^ entry via store-operated Ca^2+^ entry (SOCE) and promote cell cycle progression via Atk pathway [42]. Orai3 silencing downregulated MAPK kinase pathway via diminishing the phosphorylation form of ERK1/2, and expression of C-myc, which triggered cell cycle arrest in G1 phase in breast cancer [43]. On the contrary, overexpression of Ca^2+^ release-activated Ca^2+^ channel protein 1 (Orai1), also known as CRACM1, triggered reduction of store-operated Ca^2+^ influx and attenuation of EGF-mediated proliferative signaling and driving cell cycle arrest in A549 lung cancer cells [44]. Furthermore, antigen-stimulated opening of Ca^2+^ release activated Ca^2+^ (CRAC) channel, a highly Ca^2+^-selective store-operated channel, enables the refilling of ER Ca^2+^ stores and maintain the persistency of Ca^2+^ oscillations which are first identified essential for T cell proliferation and cytokine production [45]. Conformational change and redistribution of stromal interaction molecule 1 (STIM1), the ER Ca^2+^ sensor, and Orai1, a key subunit of CRAC channel pore are required for activation of CRAC channel, which, in turn, triggers Ca^2+^ release from ER lumen into cytosol through activating IP_3_R and/or (Ca^2+^)_e_ influx [46, 47]. Also, the depolarization-induced opening of VGCC Ca_v_1.2 is directly suppressed by STIM1, causing a sustained internalization of VGCC Ca_v_1.2 [48]. Heretofore, Wang, et al. [49] identified that STIM1 was significantly overexpressed in lung cancer tissues as compared to that of non-neoplastic lung tissues; furthermore, Ge, et al. [50] revealed that STIM1 knockdown induced cell cycle arrest at G2/M and S phases through alleviating expression of CDK1 and 2 in A549 and SK-MES-1 cells, and abolishing tumorigenesis and growth of lung cancer cells in nude mice xenograft. Upon reaching to cytosol, Ca^2+^ often forms complexes with the molecular components of “(Ca^2+^)_i_ signaling molecular toolkit” specific for each cell type given. Among such direct effectors essential for (Ca^2+^)_i_ signaling are CaM and Ca^2+^/CaM-dependent protein kinases II (CaMKII), protein phosphatase 2B (PP2B) and protein kinase C (PKC), modulating the transcriptional activity of various transcription factors for a large number of genes required for cell cycle progression [51]. Using 1-[*N*, *O*-Bis(5-isoquinolinesulfonyl)-*N*-methyl-L-tyrosyl]-4-phenylpiperazine) (KN-62), a specific CaMKII antagonist, Williams, et al. [52] observed that blockade of CaMKII activity inhibited the exponentially proliferative capacity of SCLC cells through ameliorating cell cycle arrest at S phase. Altogether, the Ca^2+^ channels/pumps/exchangers and Ca^2+^-handling proteins identified affect cell cycle progression in lung cancer cells (Table 1).

**Table 1.** The summary of the roles of the major Ca^2+^ channel/pump/exchanger and Ca^2+^-handling proteins in regulation of cell cycle in lung cancer cells

**Ca^2+^-channel/pump/exchangerand Ca^2+^-handling proteins**	**Cell line**	**Expression**	**Described roles**
Orai3	NCI-H23 and NCI-H460	↑	Decreased SOCE, abolished cell proliferation and triggered cell cycle arrest at G0/G1 phase [42]
Orai1/CRACM1	A549	Not determined	Orai1/CRACM1 overexpression attenuated EGF-mediated store-operated (Ca^2+^)_e_ influx, and triggers G0/G1 cell cycle arrest [44]
STIM1	A549 SK-MES-1	↑	STIM1 silencing inhibited colony formation, and induced cell cycle arrest at G2/M and S phases [50]
CaMKII	NCI-H69, NCI-H128, NCI-H146 and NCI-H345	Not determined	KN-62-induced inhibition of CaMKII activity triggered reduced DNA synthesis and cell cycle arrest at S phase [52]
CaNAα	SBC-3	↑	Promoted G1/S phase transition [35]

↑ : increased

### The effects of dysregulated Ca^2+^ signaling on apoptosis

Apoptosis, a programmed cell death (PCD), is important for removal of mutated or transformed cells from the body essential for embryogenesis, development and tissue homeostasis of multicellular organisms. Principally, apoptosis comprises two core pathways: (1) the extrinsic pathway and (2) the intrinsic pathway, which are sequentially referred to as death receptor (DR)-mediated pathway and mitochondrial pathway [53]. Though stimulated in different manners, these pathways commonly converge into the same destination. Both the intrinsic and extrinsic apoptotic mechanisms lead to the activation of caspase 8, an initiator of a series of the apoptotic events through activating caspase 3, 6 and 7, subsequently resulting in cell collapse, chromatin condensation, breakdown of nuclear DNA, formation of apoptotic bodies and recognition of apoptotic cells by phagocytic cells [54].

During carcinogenesis, cancer cells acquired specific mechanisms of protection against apoptosis [55]. Among these, Ca^2+^ has been emerged as an important element exploiting its specialized effects towards regulation of apoptosis [56]. Under specific conditions at the initial step of mitochondria-induced apoptosis, overload of mitochondrial Ca^2+^, a vital sensitizer of the mitochondrial permeability transition (MPT) triggers mitochondrial swelling, perturbation of the mitochondrial outer membrane [57]. MPT pore-triggered release of the pro-apoptotic factors such as cytochrome c, apoptosis inducing factor (AIF), procaspase-9, Smac/DIABLO, and endonuclease G into cytosol causes a massive activation of proteases (caspases) and phospholipases [58–61]. To resist to apoptosis, cancer cells typically acquire the highly protective mechanisms against abolishment of mitochondria-triggered Ca^2+^ signals.

ER-derived Ca^2+^ signals, critical for regulating the apoptosis-related events, are also engaged in the mitochondria-induced apoptosis via the mitochondria-associated membranes (MAMs) juxtaposed between ER and mitochondria [62, 63]. Ca^2+^ storage in the ER is accomplished by the action of SERCA and of the intraluminal ER Ca^2+^-binding proteins such as BiP, calreticulin and calnexin whereas the release of Ca^2+^ from the ER is virtually mediated by IP_3_Rs. The role of MAMs for regulation of (Ca^2+^)_i_ homeostasis is mediated by IP_3_R3, RyR and SERCA [63]. Upon ER-derived Ca^2+^ signal-induced mitochondrial remodeling, B-cell lymphoma-2 (Bcl-2) proteins the first anti-apoptotic proteins identified regulate apoptosis via regulating Ca^2+^ transfer between ER and mitochondria [64]. These proteins are functionally categorized into the anti-apoptotic group (Bcl-2, BCL-X_L_, and Mcl-1) and the pro-apoptotic group (Bax, Bak, Bim, Bid, etc.). Anti-apoptotic Bcl-2 proteins regulates apoptosis by modulating the ER-mitochondrial Ca^2+^ transfer via the MAMs [65] while overexpression of pro-apoptotic Bcl-2 decreased both ER-Ca^2+^ release either by the direct control of IP_3_R3-mediated pore opening or by lowering the Ca^2+^ content of the ER, which weakens Ca^2+^-triggered MPT, and thus enables cancer cell to resist to apoptosis [66]. Abnormal upregulation of pro-apoptotic Bcl-2 is frequently observed in various types of cancer cells such as gastric, colon, breast and lung cancer [67–70]. Indeed, alleviation of ER-Ca^2+^ levels and signals has been observed in pro-apoptotic *Bax* and *Bak*-knockout murine embryonic fibroblasts (MEFs) [71]. Strikingly, enhanced ER Ca^2+^ levels by ectopic expression of SERCA2 rescue their sensitivity to death stimuli, suggesting the functional necessity of Bcl-2 proteins in regulating the ER-mitochondrial Ca^2+^ gateway and cell death in MEFs [71]. Bcl-2 mutant has also been reported to reduce ER Ca^2+^ by inhibition of SERCA2 as a consequence of a reduction of SOCE [72, 73]. Bergner, et al. [74] reported that a reduction of Ca^2+^ content correlated with a decreased expression of SERCA2 pumping Ca^2+^ into the ER, an increased expression of IP_3_R releasing Ca^2+^ from the ER in various types of lung cancer cell lines. In contrast, the detailed mechanisms underlying Bcl-2-mediated regulation of SOCE remains controversial. Depletion of Ca^2+^ in the ER causes translocation of the SOC channel activator, STIM1, to the PM [75]. Thereafter, binding of STIM1 to Orai1 and/or transient receptor potential channel 1 (TRPC1) forces them to open for allowing Ca^2+^ entry across the PM [75].

Oncogenic K-RAS, which degenerates ER Ca^2+^ dynamics [76], and Akt, which not only phosphorylates and inactivates several pro-apoptotic Bcl-2 such as Bad, Bax and hexokinase-2, but more importantly diminishes mitochondrial Ca^2+^ overload via alleviating IP_3_R opening, also contribute towards inhibition of the intrinsic apoptosis inhibition [77, 78]. Concomitantly, downregulation of protein phosphatase and tensin homolog (PTEN) in NSCLC tumors [79] antagonized F-box and leucine rich repeat protein 2 (FBXL2)-induced ubiquitination of IP_3_R3, thereby stabilizing IP_3_R3 in ER [80]. Besides, EGFRs with its aberrant expression and constitutive activation in in NSCLC [81] stimulate three of the most well-characterized signaling branches such as Ras-MAPK, phosphoinositol 3 kinase (PI3K)-protein kinase B (PKB)/Akt and phospho lipase C (PLC)-PKC pathways, enhancing ER-mitochondria Ca^2+^ transfer, thereby abolishing mitochondria-induced apoptosis. Also, loss of promyelocytic leukemia protein (PML) isoform IV, a suppressor of transcriptional activity of EGFR, for instance, on *cyclin D1* gene promoter in lung cancer cells [82] also contributed to the EGFR-mediated mitochondria-induced apoptosis and cell cycle arrest [81].

### The effects of dysregulated Ca^2+^ signaling on metastatic lung cancer

Metastasis, a term used to describe the spread of cancer cells from the primary tumor to surrounding tissues and to distant organs, is the major cause of morbidity and mortality in cancer patients. Among all solid tumors, SCLC is one of the most aggressive malignancy associated with a majority of patients diagnosed with metastatic disease [83]. Metastatic SCLC cells easily dissociate from lungs to disseminate throughout bloodstream and/or lymph system to anatomically distant organs such as lymph nodes, brain, liver and bone [83].

Progress of metastatic cascades primarily begins with the loss of cell-extracellular microenvironment [extracellular matrix (ECM)] as well as cell-cell attachment. Cells are connected to the ECM at focal adhesion points by structural complexes linking membrane spanning integrins to the cytoskeleton. Therefore, migratory capacity of cancer cells are principally assessed by the rate of focal adhesion assembly and disassembly. Noticeably, Ca^2+^ pulses promote the association of focal adhesion kinase (FAK), which regulates focal adhesion turnover, with the focal adhesion complex (FAC). More detailed, Ca^2+^ pulses strengthen the FAK at the specific sites where it is phosphorylated in a CaMKII-dependent manner. The movement of migrating cells is initialized by the extension of the protrusive front edge, which is known as lamellipodia. For cell protrusion, actin polymerization in lamellipodia and filopodia is required [84]. The attachment of lamellipodia to the substratum and contraction of the rear edge enable cells to move towards the lamellipodia. Establishment of a gradient difference of (Ca^2+^)_i_ levels, which was lower in the front, and higher in the rear of the migrating, polarized cells, caused rear retraction, focal adhesion (at the rear) and protrusion (at the front) [85, 86]. Following protrusion, the cell front starts to retract and locally adhere to ECM in lamella [87], which plays a pivotal in actomyosin contractility and F-actin disassembly in a treadmill-like manner [88]. Furthermore, actin and myosin, two of the important structural constituents, are regulated indirectly by Ca^2+^ signaling via the activation of the cyclic element-mediated Ca^2+^-dependent kinases, named calpain [89], and regulation of Rac1, RhoA, Cdc42, protein kinase A (PKA) [90, 91], and local Ca^2+^ signals between lamellipodia and lamella [92]. For retraction of the rear edge, Ca^2+^ signaling play a vital role in maintaining contractivity and stabilizing the directional movement via modulating the Ca^2+^ influx through L-type Ca^2+^ channels [93]. Ca^2+^-dependent MLC kinase (MLCK)-mediated phosphorylation of myosin light-chain (MLC) triggers myosin II-induced actomycin contractility [94], promoting the retraction and adhesion more efficiently [95, 96].

It is well-characterized that “local Ca^2+^ pulses” in the front of migrating cells are released from ER via IP_3_-induced activation of IP_3_Rs [97], which is generated through activation of RTK-PLC-dependent signaling pathways. It is therefore proposed that ER-derived Ca^2+^ release by the axis of RTK-PLC-IP_3_R would be major source of Ca^2+^ pulses in the front of migrating cells. Indeed, EGFR, also known as HER1, belonging to the ErbB family of structurally related RTKs that comprises four isoforms: ErbB2 (HER2), ErbB3 (HER3) and ErbB4/HER4 [98], is overexpressed and constitutively activated in 62% of NSCLC cases [99]. It is clear that EGFR is the key activator of the ERK/MAPK, Akt-PI3K, and PLCγ-PKC signaling cascades [100]; furthermore, Tsai, et al. [101] reported RTK and PLC were enriched at the leading edge of migrating cells, in correlation with intensity of local Ca^2+^ pulses in the cell front.

In addition to RTK, G-protein coupled receptors (GPCRs) on local Ca^2+^ pulses in the cell front via activating PLC, which hydrolyzes phosphatidylinositol-4,5-bisphosphate (PIP_2_) to release IP_3_, which binds to IP_3_R to trigger transient Ca^2+^ release [102, 103]. In a meanwhile, depletion of ER luminal Ca^2+^ sensitizes SOC channels located at PM to infux (Ca^2+^)_e_ across PM [104]. Chantôme, et al. [105] once reported that the interaction of Orai1 with SK3 channel, a potassium channel belonging to the small conductance Ca^2+^-activated potassium (KCa) channel family, regulated the constitutive (Ca^2+^)_e_ entry through Orai1 localization within the lipid raft, which affected the migratory ability of breast cancer cells. Disruption of interaction between SK3 and Orai1 from lipid rafts weakened SK3-mediated Ca^2+^ entry, migration and bone metastasis [105]. Moreover, STIM/Orai-mediated SOCE is also essential for elevation of (Ca^2+^)_i_ level. STIM1, the ER Ca^2+^ sensor, and Orai3, constituent a native SOC entry essential for NSCLC progression [42, 50]. Increasing evidence implied that STIM1 assisted the turnover of cell matrix adhesion complexes, thereby enhancing cell migration by maintaining local Ca^2+^ pulses in the front of migrating cells [106]. In migrating cells, local Ca^2+^ pulses near its leading edge cause depletion of Ca^2+^ in the front ER, resulting in activation of STIM1 at the cell front [101]. More specifically, STIM1 was remarkably translocated to the ER-PM junction in cell front rather than cell rear during cell migration, thereby promoting maintenance of cell polarity and motility [101].

In addition, inhibition of the activity of Ca^2+^ permeable channels, PMCA and NCX, by the specific blockers, which are vanadate (V^5+^) and KB-R7943, respectively, led to a decrease in migratory capacity of MDCK-F cells, suggesting these channels were of significant importance for cell migration [107]. Furthermore, inactivation of ER membrane-located SERCA, which is responsible for pumping (Ca^2+^)_i_ into the ER lumen, triggering a leak of the ER lumical Ca^2+^ into cytosol [101]. The high (Ca^2+^)_i_ concentration caused MLCK saturation and myosin contractility [101]. Indeed, Atousa Arbabian [108] once reported that dysfunctional SERCA diminishes the ER luminal Ca^2+^, thereby disabling further Ca^2+^ signaling through IP_3_Rs, suggesting a physiological importance of SERCA on lung cancer progression, invasion and metastasis.

## Metastatic lung cancer with bone metastasis

Bone is one of the most common metastatic sites for lung cancer, in which 36% of patients with bone lesions, and a range of 20%–60% with bone marrow micrometastasis [109, 110]. Metastasis lung cancer with bone metastasis is a major source of morbidity and mortality; however, it is not frequently detected in the patients until pain, skeletal-related events (SREs) in spine, ribs, pelvis and proximal long bones, pathological fractures and nerve compression syndromes occur [111]. Therefore, comprehension of why and how the various specific features of bone microenvironment, associated with spatio-temporal fluctuations of Ca^2+^ signaling network preferentially towards bone metastasis is essential for development of the efficacious drug program.

Bone is a dynamic organ included a variety of embryo-derived cells such as hematopoietic, stromal, endothelial, adipocytes, OCs, OBs and osteocytes [112]. Two important mediators of the hematopoietic stem cell (HSC) environment are (1) the chemo-attractant stromal derived factor-1 (SDF-1) or C-X-C motif chemokine ligand 12 (CXCL12) and (2) the cell adhesion factor (Annexin2 or ANXA2) [113]. CXCL12 regulates HSC homing to the bone marrow, while ANXA2 is likely involved in HSC binding to the osteoblastic niche, and may act as an anchor of CXCL12 and aid in localization to the niche [113]. The disseminated tumor cells (DTCs) could survive in a quiescent state in bone marrow of cancer patients for years. Increasing evidence suggests that DTCs gain access to the bone marrow using homing mechanisms similar to those of HSCs. The interaction of CXCL12, which is secreted by bone marrow stromal cells including fibroblasts and endothelial cells, to C-X-C motif chemokine receptor 4 (CXCR4), which is aberrantly expressed in tumor cells, allows tumor cells to directionally migrate to bone [114] mainly through upregulating the two most crucial downstream pathways comprising IP_3_K and MAPK pathways. Nonetheless, the detailed mechanisms of how CXCR4 /CXCL12 interaction stimulates metastasis and/or tumor growth and their complete implications on metastatic lung cancer in bone are unknown.

Bone homeostasis is maintained by two major types of bone cells, consisting of OBs and OCs, which are responsible for bone formation and bone resorption, respectively [115]. Of these, OBs, differentiated from mesenchymal stem cells, participate in regulating bone remodeling by generating ECM and calcium phosphate crystals, which are deposited into the interstitial space of the matrix [116]. OCs, the polarized, multinucleated myoleoid lineage cells, adhere to the bone surface through αvβ3 integrin, form ruffled borders, and secrete acid to solubilize calcium phosphate crystals as well as secret the collagenases and proteinases such as tartrate-resistant acid phosphatase (TRAP), matrix metallopeptidase 9 (MMP9), and cathepsin K (CTSK) that demineralize and degrade extracellular proteins such as type I collagen [117].

Bone has several particular characteristics such as acidified milieu, hypoxia (O_2_ deficiency) and high level of (Ca^2+^)_e_, enabling tumor cells to establish an acidic microenvironment via production of a large amount of lactic acid, which then creates the local areas inside bone, thereby accelerating tumor cell dormancy and promotes osteolysis [118]. The release of bone resorption-derived (Ca^2+^)_e_ triggers activation of Ca^2+^-sensing receptor (CaR), a G protein-coupled receptor, on PM of tumor cells and OBs [119], OCs [120] and especially tumor cells [121], including lung adenocarcinoma [122]. Activation of Ca^2+^-sensing receptors enhances the secretion of parathyroid hormone-related peptide (PTHrP), which subsequently binds to its receptor, PTHR1, to increase receptor activator of nuclear factor kappa-B (RANK) ligand (RANKL) expression in bone marrow stromal cells, thereby promoting osteolysis [121]. Furthermore, RANKL-mediated osteoclast differentiation triggers IP_3_R-induced local Ca^2+^ release, inducing activation of one of the master transcription factors of osteoclastogenesis, the NFATc-1 [123], subsequently entering nuclei to bind to the promoters of specific genes required for osteoclast differentiation [123]. In addition, it is unknown whether bone resorption-derived (Ca^2+^)_e_ might have been responsible for activating the Ca^2+^ channels, pumps and exchangers to promote differentiation and growth of metastatic lung cancer cells (MLCCs) in bone.

**Figure 1.**
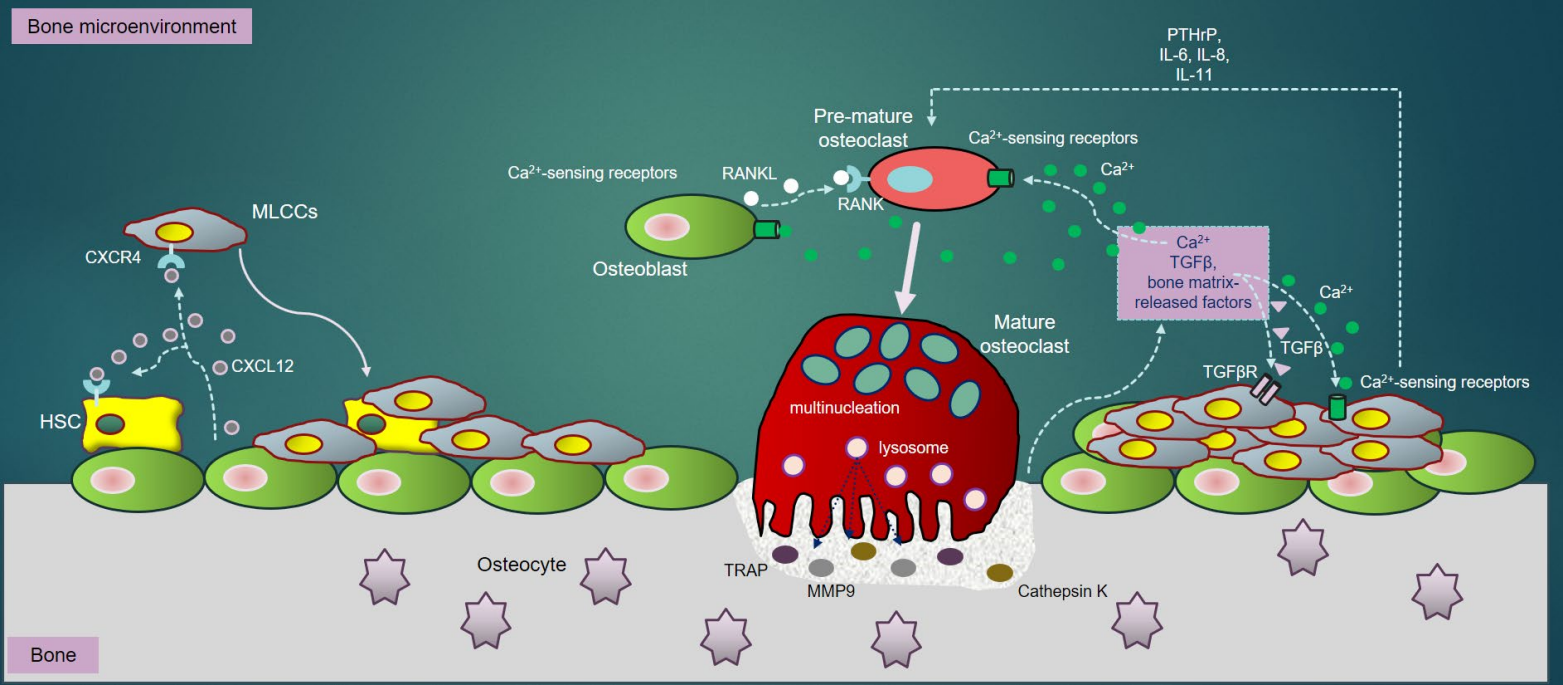
The MLCCs in bone: once in bone extracellular matrix (BEM), MLCCs encounters with the bone marrow stromal cells. The CXCR4/CXCL12 interaction enables MLCCs to attach to the osteogenic niches, strengthening MLCCs to survive, proliferate and metastasize. RANKL, secreted by OBs, directly binds to RANK receptor on PM of pre-OCs, triggering differentiation of pre-OCs into mature (multinucleated) OCs. Mature OCs secrete bone-resorbing elements including TRAP, CTSK and MMP9, all of which resorb bone to release Ca^2+^ ions, TGFβ and other bone matrix-release factors into BEM. Ca^2+^ ions released subsequently activate OC differentiation through NFATc-1 downstream signaling pathways. Besides, bone resorption-derived Ca^2+^ ions interact with Ca^2+^-sensing receptors highly expressed in OBs, OCs and MLCCs, which further promotes survival, proliferation, differentiation and metastasis of MLCCs in bone. Moreover, the interaction of bone resorption-derived TGFβ to its receptor, TGFβR highly expressed in MLCCs, activates several important downstream signaling cascades such as MAPK, PI3K/Akt, and Rho-like GTPase, which synergistically enhance metastatic properties of MLCCs in bone. Additionally, the ILs (IL-6, IL-8 and IL-11) and PTHrP secreted by MLCCs also contribute towards augmentation of OC differentiation and bone resorption

In regardless of RANKL, TGFβ, a bone resorption-derived factor, enhances the PTHrP expression in tumor cells and OBs, thereby promoting osteolysis [124]. Specifically, TGFβ-mediated signaling pathway activating a couple of important intracellular cascades, consisting of MAPK, PI3K/Akt, and Rho-like GTPase signaling cascades [125], critically acts as a driver of tumor progression and metastasis [126]. Importantly, the tumor cells could significantly produce not only the ILs such as IL-6, IL-8, and IL-11, required for osteoclastogenesis [127], but also strengthen the expression of CXCR4 and CXCR7, establishing a “fertile soil” that accelerates tumor cells to adhere to bone matrix and thrive in bone [128, 129] (Figure 1).

## Conclusion

In this article, I have reviewed the role of Ca^2+^ as a key regulator of lung cancer progression and bone metastasis with the metastatic lung cancer. Principally, Ca^2+^ signals are intrinsic to all aspects of cancer biology, especially in the metastatic lung cancer with bone metastasis. Therefore, identification of the key Ca^2+^ channels, pumps and/or exchangers would be beneficial for development of treatment strategies for lung cancer. Unfortunately, the exact mechanisms underlying Ca^2+^ signaling-mediated regulation of lung cancer progression has been incomprehensively understood. Basically, three major steps of bone metastasis required include (1) migration, (2) adhesion and invasion to bone, and (3) proliferation, growth and metastasis in bone. However, it is unclear whether aberrant changes in Ca^2+^ signals are one of the primary causes of initiation of lung cancer progression.

To what extent can therapeutic strategies exploit these Ca^2+^-regulated processes? Accumulating preclinical and clinical evidence has elucidated the relationship between aberrant Ca^2+^ signaling and tumor progression. Using the specific blockers of Ca^2+^ channels, pumps and/or exchangers has demonstrated the significant antitumor effects on lung cancer progression (Table 2), indicating that Ca^2+^ signaling would be a promising target for novel lung cancer treatments. However, before contemplating such efficacious therapeutic interventions based on pharmacological modulation of Ca^2+^-regulators, it is crucial to design more potent and specific, but less off-target drugs targeting Ca^2+^-regulators, including Ca^2+^ channels, pumps and/or exchangers. Therefore, further studies are required to verify the toxicity and pharmacokinetic of such modulators prior to the clinical tests.

**Table 2.** Summary of the major compounds targeting Ca^2+^ channels/pumps/exchangers in lung cancer progression

**Ca^2+^ channel/pump/exchanger**	**Drug Candidates**	**Pharmacological effects**
TRPCs	SKF-96365	Cell cycle arrest at S/G2M phase, and invasive ability in A549 cell line [130]
	ATRA 2-ABP	Proliferative inhibition in A549 cells line [131]
	Carvacrol	Degeneration of cell morphology, and apoptosis in A549 cell line [132, 133]
	Capsaicin	Apoptosis in SCLC cell lines, NCI-H82, NCI-H69 [134]
	Tetrahydrocannabinol and cannabidiol	Inhibition of proliferation, epithelial-mesenchymal transition (EMT) and migration in A549, H460 and H1792 lung cancer cell lines [135]
	Dexamethasone	Growth suppression in NSCLC cell lines, A549 and H1299 [136]
RyR	Compound K	ER-mediated apoptosis in A549 and SK-MES-1 cell lines [137]
	Paclitaxel	Cell cycle arrest at G2/M phase in A549 cell line [138]
IP_3_R3	Α-Lipoic acid (LA)	Apoptosis in A549 cell line [139]
	Curcumin	Apoptosis in NSCLC cell lines, A549 and H1299 [140]
VGCCs	Verapamil, Diltiazem, and Nifepine	Cell death in chemoresistant lung cancer cells derived from A549 cell line [141]
SERCA	2-deoxy D-glucose and metformin	Apoptosis in A549 cell line [142]
Voltage-dependent anion channel (VDAC)	R-Tf-D-LP4	Apoptosis and inhibition of tumor growth HepG2 and Huh-7 cell lines [143]

## Abbreviations

(Ca^2+^)_e_: extracellular Ca^2+^
(Ca^2+^)_i_: intracellular Ca^2+^
Bcl-2: B-cell lymphoma-2
CaM: calmodulin
CaMKII: calmodulin-dependent protein kinase II
CaN: calciuneurin
CRAC: Ca^2+^ release-activated Ca^2+^ channels
CXCL12: C-X-C motif chemokine ligand 12
CXCR4: C-X-C motif chemokine receptor 4
ECM: extracellular matrix
EGFR: epidermal growth factor receptor
ER/SR: endoplasmic/sarcoplasmic reticulum
ER: endoplasmic reticulum
HSCs: hematopoietic stem cells
IL: interleukin
IP_3_: inositol-1,4,5-trisphosphate
IP_3_R: IP_3_ receptor
MAMs: mitochondria-associated membranes
MAPK: mitogen-activated protein kinase
MLCC: metastatic lung cancer cell
MPT: mitochondrial permeability transition
NFAT: nuclear factor of activated T cell
NSCLC: non-small cell lung cancer
OBs: osteoblasts
OCs: osteoclasts
Orai1: Ca^2+^ release-activated Ca^2+^ channel protein 1
PI3K: phosphoinositol 3 kinase
PKC: protein kinase C
PLC: phospho lipase C
PM: plasma membrane
PTHrP: parathyroid hormone-related peptide
RANKL: receptor activator of nuclear factor kappa-B ligand
RyR: ryanodine receptor
SCLC: small lung cell lung cancer
SERCA: SR/ER Ca^2+^-ATPase
SOC: store-operated Ca^2+^ channel
SOCE: store-operated Ca^2+^ entry
STIM1: stromal interaction molecule 1
TGFβ: transforming growth factor beta
TRP: transient receptor potential
VGCC: voltage-gated Ca^2+^ channel

## Declarations

### Copyright

© The Author(s) 2021.

## References

[1] Cui C Merritt R Fu L Pan Z. Targeting calcium signaling in cancer therapy. Acta Pharm Sin B. 2017;7:3–17. 10.1016/j.apsb.2016.11.001 28119804 PMC5237760

[2] Islam MS. Calcium signaling: from basic to bedside. Adv Exp Med Biol. 2020;1131:1–6. 10.1007/978-3-030-12457-1_1 31646504

[3] Pchitskaya E Popugaeva E Bezprozvanny I. Calcium signaling and molecular mechanisms underlying neurodegenerative diseases. Cell Calcium. 2018;70:87–94. 10.1016/j.ceca.2017.06.008 28728834 PMC5748019

[4] Yoshikawa F Morita M Monkawa T Michikawa T Furuichi T Mikoshiba K. Mutational analysis of the ligand binding site of the inositol 1,4,5-trisphosphate receptor. J Biol Chem. 1996;271:18277–84. 10.1074/jbc.271.30.18277 8663526

[5] Dulhunty AF Board PG Beard NA Casarotto MG. Physiology and pharmacology of ryanodine receptor calcium release channels. Adv Pharmacol. 2017;79:287–324. 10.1016/bs.apha.2016.12.001 28528672

[6] Kania E Roest G Vervliet T Parys JB Bultynck G. IP3 receptor-mediated calcium signaling and its role in autophagy in cancer. Front Oncol. 2017;7:140. 10.3389/fonc.2017.00140 28725634 PMC5497685

[7] Di Leva F Domi T Fedrizzi L Lim D Carafoli E. The plasma membrane Ca^2+^ ATPase of animal cells: structure, function and regulation. Arch Biochem Biophys. 2008;476:65–74. 10.1016/j.abb.2008.02.026 18328800

[8] Reppel M Fleischmann BK Reuter H Sasse P Schunkert H Hescheler J. Regulation of the Na^+^/Ca^2+^ exchanger (NCX) in the murine embryonic heart. Cardiovasc Res. 2007;75:99–108. 10.1016/j.cardiores.2007.03.018 17462611

[9] Primeau JO Armanious GP Fisher ME Young HS. The sarcoendoplasmic reticulum calcium ATPase. Subcell Biochem. 2018;87:229–58. 10.1007/978-981-10-7757-9_8 29464562

[10] Dong N Shi L Wang DC Chen C Wang X. Role of epigenetics in lung cancer heterogeneity and clinical implication. Semin Cell Dev Biol. 2017;64:18–25. 10.1016/j.semcdb.2016.08.029 27575638

[11] Zhou Y Yu QF Peng AF Tong WL Liu JM Liu ZL. The risk factors of bone metastases in patients with lung cancer. Sci Rep. 2017;7:8970. 10.1038/s41598-017-09650-y 28827719 PMC5567132

[12] Reddi AH Roodman D Freeman C Mohla S. Mechanisms of tumor metastasis to the bone: challenges and opportunities. J Bone Miner Res. 2003;18:190–4. 10.1359/jbmr.2003.18.2.190 12568395

[13] Atkinson EG Delgado-Calle J. The emerging role of osteocytes in cancer in bone. JBMR Plus. 2019;3:e10186. 10.1002/jbm4.10186 30918922 PMC6419608

[14] Permyakov EA Kretsinger RH. Cell signaling, beyond cytosolic calcium in eukaryotes. J Inorg Biochem. 2009;103:77–86. 10.1016/j.jinorgbio.2008.09.006 18954910

[15] Pande G Kumar NA Manogaran PS. Flow cytometric study of changes in the intracellular free calcium during the cell cycle. Cytometry. 1996;24:55–63. 10.1002/(SICI)1097-0320(19960501)24:1<55::AID-CYTO7>3.0.CO;2-H 8723903

[16] Elangovan IM Vaz M Tamatam CR Potteti HR Reddy NM Reddy SP. FOSL1 promotes kras-induced lung cancer through amphiregulin and cell survival gene regulation. Am J Respir Cell Mol Biol. 2018;58:625–35. 10.1165/rcmb.2017-0164OC 29112457 PMC5946328

[17] Huang H Weng H Zhou H Qu L. Attacking c-Myc: targeted and combined therapies for cancer. Curr Pharm Des. 2014;20:6543–54. 10.2174/1381612820666140826153203 25341931

[18] Hernández S Hernández L Beà S Cazorla M Fernández PL Nadal A cdc25 cell cycle-activating phosphatases and c-myc expression in human non-Hodgkin’s lymphomas. Cancer Res. 1998;58:1762–7. 9563496

[19] Li Z Van Calcar S Qu C Cavenee WK Zhang MQ Ren B. A global transcriptional regulatory role for c-Myc in Burkitt’s lymphoma cells. Proc Natl Acad Sci U S A. 2003;100:8164–9. 10.1073/pnas.1332764100 12808131 PMC166200

[20] Orian A van Steensel B Delrow J Bussemaker HJ Li L Sawado T Genomic binding by the Drosophila Myc, Max, Mad/Mnt transcription factor network. Genes Dev. 2003;17:1101–14. 10.1101/gad.1066903 12695332 PMC196053

[21] Zhang Y Zhang A Shen C Zhang B Rao Z Wang R E2F1 acts as a negative feedback regulator of c-Myc-induced hTERT transcription during tumorigenesis. Oncol Rep. 2014;32:1273–80. 10.3892/or.2014.3287 24969314

[22] Zheng L Suzuki H Nakajo Y Nakano A Kato M. Regulation of c-MYC transcriptional activity by transforming growth factor-beta 1-stimulated clone 22. Cancer Sci. 2018;109:395–402. 10.1111/cas.13466 29224245 PMC5797808

[23] Rotow JK Gui P Wu W Raymond VM Lanman RB Kaye FJ Co-occurring alterations in the RAS-MAPK pathway limit response to MET inhibitor treatment in MET exon 14 skipping mutation-positive lung cancer. Clin Cancer Res. 2020;26:439–49. 10.1158/1078-0432.CCR-19-1667 31548343 PMC6980768

[24] Pradhan R Singhvi G Dubey SK Gupta G Dua K. MAPK pathway: a potential target for the treatment of non-small-cell lung carcinoma. Future Med Chem. 2019;11:793–5. 10.4155/fmc-2018-0468 30994024

[25] Asghar U Witkiewicz AK Turner NC Knudsen ES. The history and future of targeting cyclin-dependent kinases in cancer therapy. Nat Rev Drug Discov. 2015;14:130–46. 10.1038/nrd4504 25633797 PMC4480421

[26] Choi YJ Anders L. Signaling through cyclin D-dependent kinases. Oncogene. 2014;33:1890–903. 10.1038/onc.2013.137 23644662

[27] Fu H Gao H Qi X Zhao L Wu D Bai Y Aldolase A promotes proliferation and G1/S transition via the EGFR/MAPK pathway in non-small cell lung cancer. Cancer Commun (Lond). 2018;38:18. 10.1186/s40880-018-0290-3 29764507 PMC5993145

[28] Goodrich DW. The retinoblastoma tumor-suppressor gene, the exception that proves the rule. Oncogene. 2006;25:5233–43. 10.1038/sj.onc.1209616 16936742 PMC2799241

[29] Knudsen ES Pruitt SC Hershberger PA Witkiewicz AK Goodrich DW. Cell cycle and beyond: exploiting new RB1 controlled mechanisms for cancer therapy. Trends Cancer. 2019;5:308–24. 10.1016/j.trecan.2019.03.005 31174843 PMC6719339

[30] Sherr CJ. D-type cyclins. Trends Biochem Sci. 1995;20:187–90. 10.1016/s0968-0004(00)89005-2 7610482

[31] Sherr CJ Beach D Shapiro GI. Targeting CDK4 and CDK6: from discovery to therapy. Cancer Discov. 2016;6:353–67. 10.1158/2159-8290.CD-15-0894 26658964 PMC4821753

[32] Zhou YL Xu YJ Qiao CW. MiR-34c-3p suppresses the proliferation and invasion of non-small cell lung cancer (NSCLC) by inhibiting PAC1/MAPK pathway. Int J Clin Exp Pathol. 2015;8:6312–22. 26261507 PMC4525841

[33] Gupta R Dastane AM Forozan F Riley-Portuguez A Chung F Lopategui J Evaluation of EGFR abnormalities in patients with pulmonary adenocarcinoma: the need to test neoplasms with more than one method. Mod Pathol. 2009;22:128–33. 10.1038/modpathol.2008.182 18997733

[34] Goshima T Habara M Maeda K Hanaki S Kato Y Shimada M. Calcineurin regulates cyclin D1 stability through dephosphorylation at T286. Scientific Reports. 2019;9:12779. 10.1038/s41598-019-48976-7 31484966 PMC6726757

[35] Liu Y Zhang Y Min J Liu LL Ma NQ Feng YM Calcineurin promotes proliferation, migration, and invasion of small cell lung cancer. Tumour Biol. 2010;31:199–207. 10.1007/s13277-010-0031-y 20422345

[36] Oetjen E Thoms KM Laufer Y Pape D Blume R Li P The immunosuppressive drugs cyclosporin A and tacrolimus inhibit membrane depolarization-induced CREB transcriptional activity at the coactivator level. Br J Pharmacol. 2005;144:982–93. 10.1038/sj.bjp.0706127 15711594 PMC1576078

[37] Zhang Y Baumgrass R Schutkowski M Fischer G. Branches on the alpha-C atom of cyclosporin A residue 3 result in direct calcineurin inhibition and rapid cyclophilin 18 binding. Chembiochem. 2004;5:1006–9. 10.1002/cbic.200400020 15239062

[38] Chang KT Berg DK. Voltage-gated channels block nicotinic regulation of CREB phosphorylation and gene expression in neurons. Neuron. 2001;32:855–65. 10.1016/s0896-6273(01)00516-5 11738031

[39] Kahl CR Means AR. Calcineurin regulates cyclin D1 accumulation in growth-stimulated fibroblasts. Mol Biol Cell. 2004;15:1833–42. 10.1091/mbc.e03-10-0730 14767060 PMC379279

[40] Tomono M Toyoshima K Ito M Amano H Kiss Z. Inhibitors of calcineurin block expression of cyclins A and E induced by fibroblast growth factor in Swiss 3T3 fibroblasts. Arch Biochem Biophys. 1998;353:374–8. 10.1006/abbi.1998.0667 9606972

[41] Feske S Rao A Hogan PG. The Ca^2+^-calcineurin-NFAT signalling pathway. In: New Comprehensive Biochemistry. Amsterdam: Elsevier; 2007.p.365–401.

[42] Ay AS Benzerdjeb N Sevestre H Ahidouch A Ouadid-Ahidouch H. Orai3 constitutes a native store-operated calcium entry that regulates non small cell lung adenocarcinoma cell proliferation. PLoS One. 2013;8:e72889. 10.1371/journal.pone.0072889 24058448 PMC3772818

[43] Faouzi M Kischel P Hague F Ahidouch A Benzerdjeb N Sevestre H ORAI3 silencing alters cell proliferation and cell cycle progression via c-myc pathway in breast cancer cells. Biochim Biophys Acta. 2013;1833:752–60. 10.1016/j.bbamcr.2012.12.009 23266555

[44] Hou MF Kuo HC Li JH Wang YS Chang CC Chen KC Orai1/CRACM1 overexpression suppresses cell proliferation via attenuation of the store-operated calcium influx-mediated signalling pathway in A549 lung cancer cells. Biochim Biophys Acta. 2011;1810:1278–84. 10.1016/j.bbagen.2011.07.001 21782006

[45] Waite JC Vardhana S Shaw PJ Jang JE McCarl CA Cameron TO Interference with Ca^2+^ release activated Ca^2+^ (CRAC) channel function delays T-cell arrest *in vivo*. Eur J Immunol. 2013;43:3343–54. 10.1002/eji.201243255 23939929 PMC3924891

[46] Harraz OF Altier C. STIM1-mediated bidirectional regulation of Ca^2+^ entry through voltage-gated calcium channels (VGCC) and calcium-release activated channels (CRAC). Front Cell Neurosci. 2014;8:43. 10.3389/fncel.2014.00043 24605083 PMC3932444

[47] Lunz V Romanin C Frischauf I. STIM1 activation of Orai1. Cell Calcium. 2019;77:29–38. 10.1016/j.ceca.2018.11.009 30530091 PMC7617211

[48] Park CY Shcheglovitov A Dolmetsch R. The CRAC channel activator STIM1 binds and inhibits L-type voltage-gated calcium channels. Science. 2010;330:101–5. 10.1126/science.1191027 20929812

[49] Wang Y Wang H Li L Li J Pan T Zhang D Elevated expression of STIM1 is involved in lung tumorigenesis. Oncotarget. 2016;7:86584–93. 10.18632/oncotarget.13359 27863410 PMC5349937

[50] Ge C Zeng B Li R Li Z Fu Q Wang W Knockdown of STIM1 expression inhibits non-small-cell lung cancer cell proliferation *in vitro* and in nude mouse xenografts. Bioengineered. 2019;10:425–36. 10.1080/21655979.2019.1669518 31564210 PMC6779409

[51] Roderick HL Cook SJ. Ca^2+^ signalling checkpoints in cancer: remodelling Ca^2+^ for cancer cell proliferation and survival. Nat Rev Cancer. 2008;8:361–75. 10.1038/nrc2374 18432251

[52] Williams CL Phelps SH Porter RA. Expression of Ca^2+^/calmodulin-dependent protein kinase types II and IV, and reduced DNA synthesis due to the Ca^2+^/calmodulin-dependent protein kinase inhibitor KN-62 (1-[*N*,*O*-bis(5-isoquinolinesulfonyl)-*N*-methyl-L-tyrosyl]-4-phenyl piperazine) in small cell lung carcinoma. Biochem Pharmacol. 1996;51:707–15. 10.1016/s0006-2952(95)02393-3 8615909

[53] Hamada K Miyatake H Terauchi A Mikoshiba K. IP_3_-mediated gating mechanism of the IP(3) receptor revealed by mutagenesis and X-ray crystallography. Proc Natl Acad Sci U S A. 2017;114:4661–6. 10.1073/pnas.1701420114 28416699 PMC5422816

[54] Taylor RC Cullen SP Martin SJ. Apoptosis: controlled demolition at the cellular level. Nat Rev Mol Cell Biol. 2008;9:231–41. 10.1038/nrm2312 18073771

[55] Reed JC. Apoptosis mechanisms: implications for cancer drug discovery. Oncology (Williston Park). 2004;18:11–20. 15651172

[56] Orrenius S Zhivotovsky B Nicotera P. Regulation of cell death: the calcium-apoptosis link. Nat Rev Mol Cell Biol. 2003;4:552–65. 10.1038/nrm1150 12838338

[57] Rimessi A Bonora M Marchi S Patergnani S Marobbio CM Lasorsa FM Perturbed mitochondrial Ca^2+^ signals as causes or consequences of mitophagy induction. Autophagy. 2013;9:1677–86. 10.4161/auto.24795 24121707

[58] Arnoult D Gaume B Karbowski M Sharpe JC Cecconi F Youle RJ. Mitochondrial release of AIF and EndoG requires caspase activation downstream of Bax/Bak-mediated permeabilization. EMBO J. 2003;22:4385–99. 10.1093/emboj/cdg423 12941691 PMC202365

[59] Liu J Yoshikawa H Nakajima Y Tasaka K. Involvement of mitochondrial permeability transition and caspase-9 activation in dimethyl sulfoxide-induced apoptosis of EL-4 lymphoma cells. Int Immunopharmacol. 2001;1:63–74. 10.1016/s1567-5769(00)00016-3 11367518

[60] Petronilli V Penzo D Scorrano L Bernardi P Di Lisa F. The mitochondrial permeability transition, release of cytochrome c and cell death. Correlation with the duration of pore openings *in situ*. J Biol Chem. 2001;276:12030–4. 10.1074/jbc.M010604200 11134038

[61] Zhou LL Zhou LY Luo KQ Chang DC. Smac/DIABLO and cytochrome c are released from mitochondria through a similar mechanism during UV-induced apoptosis. Apoptosis. 2005;10:289–99. 10.1007/s10495-005-0803-9 15843890

[62] Kerkhofs M Bittremieux M Morciano G Giorgi C Pinton P Parys JB Emerging molecular mechanisms in chemotherapy: Ca^2+^ signaling at the mitochondria-associated endoplasmic reticulum membranes. Cell Death Dis. 2018;9:334. 10.1038/s41419-017-0179-0 29491433 PMC5832420

[63] Missiroli S Danese A Iannitti T Patergnani S Perrone M Previati M Endoplasmic reticulum-mitochondria Ca^2+^ crosstalk in the control of the tumor cell fate. Biochim Biophys Acta Mol Cell Res. 2017;1864:858–64. 10.1016/j.bbamcr.2016.12.024 28064002

[64] D’Orsi B Mateyka J Prehn JHM. Control of mitochondrial physiology and cell death by the Bcl-2 family proteins Bax and Bok. Neurochem Int. 2017;109:162–70. 10.1016/j.neuint.2017.03.010 28315370

[65] Vervliet T Clerix E Seitaj B Ivanova H Monaco G Bultynck G. Modulation of Ca^2+^ signaling by anti-apoptotic B-cell lymphoma 2 proteins at the endoplasmic reticulum-mitochondrial interface. Front Oncol. 2017;7:75. 10.3389/fonc.2017.00075 28516063 PMC5413508

[66] Avalle L Camporeale A Morciano G Caroccia N Ghetti E Orecchia V STAT3 localizes to the ER, acting as a gatekeeper for ER-mitochondrion Ca^2+^ fluxes and apoptotic responses. Cell Death Differ. 2019;26:932–42. 10.1038/s41418-018-0171-y 30042492 PMC6214529

[67] Kaiser U Schilli M Haag U Neumann K Kreipe H Kogan E Expression of bcl-2--protein in small cell lung cancer. Lung Cancer. 1996;15:31–40. 10.1016/0169-5002(96)00568-5 8865121

[68] Kønig SM Rissler V Terkelsen T Lambrughi M Papaleo E. Alterations of the interactome of Bcl-2 proteins in breast cancer at the transcriptional, mutational and structural level. PLoS Comput Biol. 2019;15:e1007485. 10.1371/journal.pcbi.1007485 31825969 PMC6927658

[69] Sinicrope FA Ruan SB Cleary KR Stephens LC Lee JJ Levin B. bcl-2 and p53 oncoprotein expression during colorectal tumorigenesis. Cancer Res. 1995;55:237–41. 7812951

[70] Tao S Gu J Wang Q Zheng L. Translational control of Bcl-2 promotes apoptosis of gastric carcinoma cells. BMC Cancer. 2021;21:12. 10.1186/s12885-020-07711-6 33402109 PMC7786514

[71] Scorrano L Oakes SA Opferman JT Cheng EH Sorcinelli MD Pozzan T BAX and BAK regulation of endoplasmic reticulum Ca^2+^: a control point for apoptosis. Science. 2003;300:135–9. 10.1126/science.1081208 12624178

[72] Chiu WT Chang HA Lin YH Lin YS Chang HT Lin HH Bcl-2 regulates store-operated Ca^2+^ entry to modulate ER stress-induced apoptosis. Cell Death Discov. 2018;4:37. 10.1038/s41420-018-0039-4 PMC5841437 29531834

[73] Dremina ES Sharov VS Kumar K Zaidi A Michaelis EK Schöneich C. Anti-apoptotic protein Bcl-2 interacts with and destabilizes the sarcoplasmic/endoplasmic reticulum Ca^2+^-ATPase (SERCA). Biochem J. 2004;383:361–70. 10.1042/BJ20040187 15245329 PMC1134078

[74] Bergner A Kellner J Tufman A Huber RM. Endoplasmic reticulum Ca^2+^-homeostasis is altered in small and non-small cell lung cancer cell lines. J Exp Clin Cancer Res. 2009;28:25. 10.1186/1756-9966-28-25 19236728 PMC2653468

[75] Luik RM Wang B Prakriya M Wu MM Lewis RS. Oligomerization of STIM1 couples ER calcium depletion to CRAC channel activation. Nature. 2008;454:538–42. 10.1038/nature07065 18596693 PMC2712442

[76] Pierro C Cook SJ Foets TCF Bootman MD Roderick HL. Oncogenic K-Ras suppresses IP3-dependent Ca^2+^ release through remodelling of the isoform composition of IP3Rs and ER luminal Ca^2+^ levels in colorectal cancer cell lines. J Cell Sci. 2014;127:1607–19. 10.1242/jcs.141408 24522186

[77] Dai Y Jin S Li X Wang D. The involvement of Bcl-2 family proteins in AKT-regulated cell survival in cisplatin resistant epithelial ovarian cancer. Oncotarget. 2017;8:1354–68. 10.18632/oncotarget.13817 27935869 PMC5352061

[78] Gottlob K Majewski N Kennedy S Kandel E Robey RB Hay N. Inhibition of early apoptotic events by Akt/PKB is dependent on the first committed step of glycolysis and mitochondrial hexokinase. Genes Dev. 2001;15:1406–18. 10.1101/gad.889901 11390360 PMC312709

[79] Yanagawa N Leduc C Kohler D Saieg MA John T Sykes J Loss of phosphatase and tensin homolog protein expression is an independent poor prognostic marker in lung adenocarcinoma. J Thorac Oncol. 2012;7:1513–21. 10.1097/JTO.0b013e3182641d4f 22982652

[80] Bononi A Bonora M Marchi S Missiroli S Poletti F Giorgi C Identification of PTEN at the ER and MAMs and its regulation of Ca^2+^ signaling and apoptosis in a protein phosphatase-dependent manner. Cell Death Differ. 2013;20:1631–43. 10.1038/cdd.2013.77 23811847 PMC3824603

[81] Hsu PC Jablons DM Yang CT You L. Epidermal growth factor receptor (EGFR) pathway, yes-associated protein (YAP) and the regulation of programmed death-ligand 1 (PD-L1) in non-small cell lung cancer (NSCLC). Int J Mol Sci. 2019;20:3821. 10.3390/ijms20153821 PMC6695603 31387256

[82] Kuo HY Chen YC Chang HY Jeng JC Lin EH Pan CM The PML isoform IV is a negative regulator of nuclear EGFR’s transcriptional activity in lung cancer. Carcinogenesis. 2013;34:1708–16. 10.1093/carcin/bgt109 23563092

[83] Ko J Winslow MM Sage J. Mechanisms of small cell lung cancer metastasis. EMBO Mol Med. 2021;13:e13122. 10.15252/emmm.202013122 33296145 PMC7799359

[84] Isogai T van der Kammen R Leyton-Puig D Kedziora KM Jalink K Innocenti M. Initiation of lamellipodia and ruffles involves cooperation between mDia1 and the Arp2/3 complex. J Cell Sci. 2015;128:3796–810. 10.1242/jcs.176768 26349808

[85] Brundage RA Fogarty KE Tuft RA Fay FS. Calcium gradients underlying polarization and chemotaxis of eosinophils. Science. 1991;254:703–6. 10.1126/science.1948048 1948048

[86] Giannone G Rondé P Gaire M Beaudouin J Haiech J Ellenberg J Calcium rises locally trigger focal adhesion disassembly and enhance residency of focal adhesion kinase at focal adhesions. J Biol Chem. 2004;279:28715–23. 10.1074/jbc.M404054200 15102844

[87] Webb DJ Parsons JT Horwitz AF. Adhesion assembly, disassembly and turnover in migrating cells--over and over and over again. Nat Cell Biol. 2002;4:E97–100. 10.1038/ncb0402-e97 11944043

[88] Burnette DT Manley S Sengupta P Sougrat R Davidson MW Kachar B A role for actin arcs in the leading-edge advance of migrating cells. Nat Cell Biol. 2011;13:371–81. 10.1038/ncb2205 21423177 PMC3646481

[89] Cortesio CL Boateng LR Piazza TM Bennin DA Huttenlocher A. Calpain-mediated proteolysis of paxillin negatively regulates focal adhesion dynamics and cell migration. J Biol Chem. 2011;286:9998–10006. 10.1074/jbc.M110.187294 21270128 PMC3060554

[90] Nobes CD Hall A. Rho GTPases control polarity, protrusion, and adhesion during cell movement. J Cell Biol. 1999;144:1235–44. 10.1083/jcb.144.6.1235 10087266 PMC2150589

[91] Tkachenko E Sabouri-Ghomi M Pertz O Kim C Gutierrez E Machacek M Protein kinase A governs a RhoA-RhoGDI protrusion-retraction pacemaker in migrating cells. Nat Cell Biol. 2011;13:660–7. 10.1038/ncb2231 21572420 PMC3746034

[92] Tsai FC Meyer T. Ca^2+^ pulses control local cycles of lamellipodia retraction and adhesion along the front of migrating cells. Curr Biol. 2012;22:837–42. 10.1016/j.cub.2012.03.037 22521790 PMC3503311

[93] Yang S Huang XY. Ca^2+^ influx through L-type Ca^2+^ channels controls the trailing tail contraction in growth factor-induced fibroblast cell migration. J Biol Chem. 2005;280:27130–7. 10.1074/jbc.M501625200 15911622

[94] Kim Y Chang S. Modulation of actomyosin contractility by myosin light chain phosphorylation/dephosphorylation through Rho GTPases signaling specifies axon formation in neurons. Biochem Biophys Res Commun. 2004;318:579–87. 10.1016/j.bbrc.2004.04.068 15120639

[95] Giannone G Dubin-Thaler BJ Rossier O Cai Y Chaga O Jiang G Lamellipodial actin mechanically links myosin activity with adhesion-site formation. Cell. 2007;128:561–75. 10.1016/j.cell.2006.12.039 17289574 PMC5219974

[96] Ofer N Mogilner A Keren K. Actin disassembly clock determines shape and speed of lamellipodial fragments. Proc Natl Acad Sci U S A. 2011;108:20394–9. 10.1073/pnas.1105333108 22159033 PMC3251093

[97] Taylor CW Taufiq-Ur-Rahman Pantazaka E. Targeting and clustering of IP3 receptors: key determinants of spatially organized Ca^2+^ signals. Chaos. 2009;19:037102. 10.1063/1.3127593 19798811

[98] Pines G Köstler WJ Yarden Y. Oncogenic mutant forms of EGFR: lessons in signal transduction and targets for cancer therapy. FEBS Lett. 2010;584:2699–706. 10.1016/j.febslet.2010.04.019 20388509 PMC2892754

[99] Engelman JA Jänne PA. Mechanisms of acquired resistance to epidermal growth factor receptor tyrosine kinase inhibitors in non-small cell lung cancer. Clin Cancer Res. 2008;14:2895–9. 10.1158/1078-0432.CCR-07-2248 18483355

[100] Wee P Wang Z. Epidermal growth factor receptor cell proliferation signaling pathways. Cancers (Basel). 2017;9:52. 10.3390/cancers9050052 PMC5447962 28513565

[101] Tsai FC Seki A Yang HW Hayer A Carrasco S Malmersjö S A polarized Ca^2+^, diacylglycerol and STIM1 signalling system regulates directed cell migration. Nat Cell Biol. 2014;16:133–44. 10.1038/ncb2906 24463606 PMC3953390

[102] Bartlett PJ Cloete I Sneyd J Thomas AP. IP3-dependent Ca^2+^ oscillations switch into a dual oscillator mechanism in the presence of PLC-linked hormones. iScience. 2020;23:101062. 10.1016/j.isci.2020.101062 32353764 PMC7191650

[103] Kiselyov K Shin DM Muallem S. Signalling specificity in GPCR-dependent Ca^2+^ signalling. Cell Signal. 2003;15:243–53. 10.1016/s0898-6568(02)00074-8 12531423

[104] Huertas MA Smith GD. The dynamics of luminal depletion and the stochastic gating of Ca^2+^-activated Ca^2+^ channels and release sites. J Theor Biol. 2007;246:332–54. 10.1016/j.jtbi.2007.01.003 17286986

[105] Chantôme A Potier-Cartereau M Clarysse L Fromont G Marionneau-Lambot S Guéguinou M Pivotal role of the lipid Raft SK3-Orai1 complex in human cancer cell migration and bone metastases. Cancer Res. 2013;73:4852–61. 10.1158/0008-5472.CAN-12-4572 23774210

[106] White C. The regulation of tumor cell invasion and metastasis by endoplasmic reticulum-to-mitochondrial Ca^2+^ transfer. Front Oncol. 2017;7:171. 10.3389/fonc.2017.00171 28848710 PMC5554129

[107] Dreval V Dieterich P Stock C Schwab A. The role of Ca^2+^ transport across the plasma membrane for cell migration. Cell Physiol Biochem. 2005;16:119–26. 10.1159/000087738 16121040

[108] Arbabian A Brouland JP Apáti Á Pászty K Hegedűs L Enyedi Á Modulation of endoplasmic reticulum calcium pump expression during lung cancer cell differentiation. FEBS J. 2013;280:5408–18. 10.1111/febs.12064 23157274

[109] Coello MC Luketich JD Litle VR Godfrey TE. Prognostic significance of micrometastasis in non-small-cell lung cancer. Clin Lung Cancer. 2004;5:214–25. 10.3816/CLC.2004.n.002 14967073

[110] Roato I. Bone metastases: when and how lung cancer interacts with bone. World J Clin Oncol. 2014;5: 149–55. 10.5306/wjco.v5.i2.149 24829862 PMC4014787

[111] Oyewumi MO Alazizi A Wehrung D Manochakian R Safadi FF. Emerging lung cancer therapeutic targets based on the pathogenesis of bone metastases. Int J Cell Biol. 2014;2014:236246. 10.1155/2014/236246 25197279 PMC4147348

[112] Weilbaecher KN Guise TA McCauley LK. Cancer to bone: a fatal attraction. Nat Rev Cancer. 2011;11:411–25. 10.1038/nrc3055 21593787 PMC3666847

[113] Jung Y Shiozawa Y Wang J Patel LR Havens AM Song J Annexin-2 is a regulator of stromal cell-derived factor-1/CXCL12 function in the hematopoietic stem cell endosteal niche. Exp Hematol. 2011;39:151–66.e1. 10.1016/j.exphem.2010.11.007 21108988 PMC3026087

[114] Reid JC Tanasijevic B Golubeva D Boyd AL Porras DP Collins TJ CXCL12/CXCR4 signaling enhances human PSC-derived hematopoietic progenitor function and overcomes early *in vivo* transplantation failure. Stem Cell Reports. 2018;10:1625–41. 10.1016/j.stemcr.2018.04.003 29742393 PMC5995456

[115] Hadjidakis DJ Androulakis II. Bone remodeling. Ann N Y Acad Sci. 2006;1092:385–96. 10.1196/annals.1365.035 17308163

[116] Ohgushi H. Osteogenically differentiated mesenchymal stem cells and ceramics for bone tissue engineering. Expert Opin Biol Ther. 2014;14:197–208. 10.1517/14712598.2014.866086 24308323

[117] Lerner UH Kindstedt E Lundberg P. The critical interplay between bone resorbing and bone forming cells. J Clin Periodontol. 2019;46 Suppl 21:33–51. 10.1111/jcpe.13051 30623989

[118] Kuchimaru T Hoshino T Aikawa T Yasuda H Kobayashi T Kadonosono T Bone resorption facilitates osteoblastic bone metastatic colonization by cooperation of insulin-like growth factor and hypoxia. Cancer Sci. 2014;105:553–9. 10.1111/cas.12391 24597654 PMC4317828

[119] Dvorak-Ewell MM Chen TH Liang N Garvey C Liu B Tu C Osteoblast extracellular Ca^2+^-sensing receptor regulates bone development, mineralization, and turnover. J Bone Miner Res. 2011;26:2935–47. 10.1002/jbmr.520 21956637 PMC3222747

[120] Yoshida N Sato T Kobayashi K Okada Y. High extracellular Ca^2+^ and Ca^2+^-sensing receptor agonists activate nonselective cation conductance in freshly isolated rat osteoclasts. Bone. 1998;22:495–501. 10.1016/s8756-3282(98)00038-6 9600783

[121] Sanders JL Chattopadhyay N Kifor O Yamaguchi T Butters RR Brown EM. Extracellular calcium-sensing receptor expression and its potential role in regulating parathyroid hormone-related peptide secretion in human breast cancer cell lines. Endocrinology. 2000;141:4357–64. 10.1210/endo.141.12.7849 11108243

[122] Wen L Sun L Xi Y Chen X Xing Y Sun W Expression of calcium sensing receptor and E-cadherin correlated with survival of lung adenocarcinoma. Thorac Cancer. 2015;6:754–60. 10.1111/1759-7714.12255 26557914 PMC4632928

[123] Song I Kim JH Kim K Jin HM Youn BU Kim N. Regulatory mechanism of NFATc1 in RANKL-induced osteoclast activation. FEBS Letters. 2009;583:2435–40. 10.1016/j.febslet.2009.06.047 19576893

[124] Johnson RW Nguyen MP Padalecki SS Grubbs BG Merkel AR Oyajobi BO TGF-beta promotion of Gli2-induced expression of parathyroid hormone-related protein, an important osteolytic factor in bone metastasis, is independent of canonical Hedgehog signaling. Cancer Res. 2011;71:822–31. 10.1158/0008-5472.CAN-10-2993 21189326 PMC3077118

[125] Smith AL Robin TP Ford HL. Molecular pathways: targeting the TGF-β pathway for cancer therapy. Clin Cancer Res. 2012;18:4514–21. 10.1158/1078-0432.CCR-11-3224 22711703

[126] Gu S Feng XH. TGF-β signaling in cancer. Acta Biochim Biophys Sin (Shanghai). 2018;50:941–9. 10.1093/abbs/gmy092 30165534

[127] Yin JJ Pollock CB Kelly K. Mechanisms of cancer metastasis to the bone. Cell Res. 2005;15:57–62. 10.1038/sj.cr.7290266 15686629

[128] Salcedo R Oppenheim JJ. Role of chemokines in angiogenesis: CXCL12/SDF-1 and CXCR4 interaction, a key regulator of endothelial cell responses. Microcirculation. 2003;10:359–70. 10.1038/sj.mn.7800200 12851652

[129] Wang J Shiozawa Y Wang J Wang Y Jung Y Pienta KJ The role of CXCR7/RDC1 as a chemokine receptor for CXCL12/SDF-1 in prostate cancer. J Biol Chem. 2008;283:4283–94. 10.1074/jbc.M707465200 18057003

[130] Yang LL Liu BC Lu XY Yan Y Zhai YJ Bao Q Inhibition of TRPC6 reduces non-small cell lung cancer cell proliferation and invasion. Oncotarget. 2017;8:5123–34. 10.18632/oncotarget.14034 28030826 PMC5341750

[131] Jiang HN Zeng B Zhang Y Daskoulidou N Fan H Qu JM Involvement of TRPC channels in lung cancer cell differentiation and the correlation analysis in human non-small cell lung cancer. PloS one. 2013;8:e67637. 10.1371/journal.pone.0067637 23840757 PMC3695899

[132] Khan I Bahuguna A Kumar P Bajpai VK Kang SC. *In vitro* and *in vivo* antitumor potential of carvacrol nanoemulsion against human lung adenocarcinoma A549 cells via mitochondrial mediated apoptosis. Sci Rep. 2018;8:144. 10.1038/s41598-017-18644-9 29317755 PMC5760660

[133] Koparal AT Zeytinoglu M. Effects of carvacrol on a human non-small cell lung cancer (NSCLC) cell line, A549. Cytotechnology. 2003;43:149–54. 10.1023/b:cyto.0000039917.60348.45 19003220 PMC3449592

[134] Lau JK Brown KC Dom AM Witte TR Thornhill BA Crabtree CM Capsaicin induces apoptosis in human small cell lung cancer via the TRPV6 receptor and the calpain pathway. Apoptosis. 2014;19:1190–201. 10.1007/s10495-014-1007-y 24878626 PMC4072851

[135] Milian L Mata M Alcacer J Oliver M Sancho-Tello M Martín de Llano JJ Cannabinoid receptor expression in non-small cell lung cancer. Effectiveness of tetrahydrocannabinol and cannabidiol inhibiting cell proliferation and epithelial-mesenchymal transition *in vitro*. PloS one. 2020;15:e0228909. 10.1371/journal.pone.0228909 32049991 PMC7015420

[136] Wang LJ Li J Hao FR Yuan Y Li JY Lu W Dexamethasone suppresses the growth of human non-small cell lung cancer via inducing estrogen sulfotransferase and inactivating estrogen. Acta pharmacol Sin. 2016;37:845–56. 10.1038/aps.2016.39 27133297 PMC4954770

[137] Shin DH Leem DG Shin JS Kim JI Kim KT Choi SY Compound K induced apoptosis via endoplasmic reticulum Ca^2+^ release through ryanodine receptor in human lung cancer cells. J Ginseng Res. 2018;42:165–74. 10.1016/j.jgr.2017.01.015 29719463 PMC5926506

[138] Roussel E Bélanger MM Couet J. G2/M blockade by paclitaxel induces caveolin-1 expression in A549 lung cancer cells: caveolin-1 as a marker of cytotoxicity. Anticancer Drugs. 2004;15:961–7. 10.1097/00001813-200411000-00005 15514565

[139] Choi SY Yu JH Kim H. Mechanism of alpha-lipoic acid-induced apoptosis of lung cancer cells. Ann N Y Acad Sci. 2009;1171:149–55. 10.1111/j.1749-6632.2009.04708.x 19723049

[140] Xu X Chen D Ye B Zhong F Chen G. Curcumin induces the apoptosis of non-small cell lung cancer cells through a calcium signaling pathway. Int J Mol Med. 2015;35:1610–6. 10.3892/ijmm.2015.2167 25847862

[141] Wong BS Chiu LY Tu DG Sheu GT Chan TT. Anticancer effects of antihypertensive L-type calcium channel blockers on chemoresistant lung cancer cells via autophagy and apoptosis. Cancer Manag Res. 2020;12:1913–27. 10.2147/CMAR.S228718 32214849 PMC7078713

[142] Hou XB Li TH Ren ZP Liu Y. Combination of 2-deoxy d-glucose and metformin for synergistic inhibition of non-small cell lung cancer: a reactive oxygen species and P-p38 mediated mechanism. Biomed Pharmacother. 2016;84:1575–84. 10.1016/j.biopha.2016.10.037 27825799

[143] Shteinfer-Kuzmine A Amsalem Z Arif T Zooravlov A Shoshan-Barmatz V. Selective induction of cancer cell death by VDAC1-based peptides and their potential use in cancer therapy. Mol Oncol. 2018;12:1077–103. 10.1002/1878-0261.12313 29698587 PMC6026870

